# Short Tandem Repeat analysis after Whole Genome Amplification of single B-lymphoblastoid cells

**DOI:** 10.1038/s41598-018-19509-5

**Published:** 2018-01-19

**Authors:** Lieselot Deleye, Ann-Sophie Vander Plaetsen, Jana Weymaere, Dieter Deforce, Filip Van Nieuwerburgh

**Affiliations:** Laboratory of Pharmaceutical Biotechnology, Ghent University, Ottergemsesteenweg 460, 9000 Ghent, Belgium

## Abstract

To allow multiple genetic analyses on a single cell, whole genome amplification (WGA) is required. Unfortunately, studies comparing different WGA methods for downstream human identification Short Tandem Repeat (STR) analysis remain absent. Therefore, the aim of this work was to assess the performance of four commercially available WGA kits for downstream human identification STR profiling on a B-lymphoblastoid cell line. The performance was assessed using an input of one or three micromanipulated cells. REPLI-g showed a very low dropout rate, as it was the only WGA method in this study that could provide a complete STR profile in some of its samples. Although Ampli1, DOPlify and PicoPLEX did not detect all selected STR markers, they seem suitable for genetic identification in single-cell applications.

## Introduction

Whole genome amplification (WGA) is inevitable to allow multiple genetic analyses on either a single cell or a limited number of cells. WGA increases the amount of input-DNA from pg-level up to ng- or µg-level. However, it is well known that several WGA methods introduce bias during amplification and thereby influence the results of downstream genetic analyses^1,2^. The nature of the introduced bias depends on the WGA method that is used. Therefore, a WGA method must be selected according to the required downstream application. Some methods will result in a non-uniform coverage, while others will introduce more nucleotide errors^3^. Recently, many researchers have studied the effect of WGA on copy number variant (CNV) analysis^4–8^ and targeted genotyping analysis^9^. However, studies comparing different WGA methods for human identification Short Tandem Repeat (STR) analysis of a single cell or a limited number of cells remain absent.

The growing interest in single-cell analysis for clinical applications raises the need to identify single cells. Such a genetic analysis that is often performed on only a limited number of cells is STR analysis for human identification DNA profiling. STRs are short DNA-fragments with different lengths (between 100 and 500 base pairs) within the population. A multiplex-PCR is executed to analyze a standard set of STR loci and to create an STR profile. Except for identical twins, each person has a unique STR profile. Therefore, single-cell DNA profiling is particularly valuable in the context of cell-based non-invasive prenatal testing (cbNIPT). As no specific fetal cell marker has been discovered so far, after the isolation of a single fetal cell from a maternal blood sample, the fetal identity of this cell needs to be confirmed prior to further downstream procedures. In this way, maternal cell contamination in the sample can be excluded. Other clinical applications, such as preimplantation genetic diagnosis (PGD), might also apply STR analysis to assure the nature of the isolated cells^10^. After fetal confirmation, genetic analysis such as CNV analysis is performed on this unique cell. Hence, to allow both STR and CNV analysis on a single cell, WGA is required.

Unfortunately, a WGA method that is ideal for STR genotyping might be less suitable for CNV analysis. For example, previous research by Denis *et al*. demonstrates that multiple displacement amplification (MDA) WGA might be applicable for STR analysis^10^. In contrast, MDA is not considered the most preferable WGA method for CNV analysis^6^. The aim of this study was to assess the performance of four commercially available WGA kits for downstream human identification STR on a B-lymphoblastoid cell line. REPLI-g Single Cell Kit, PicoPLEX WGA Kit, Ampli1 WGA Kit and DOPlify WGA were compared in parallel for their ability to produce WGA product, adequate for human identification STR analysis. The same WGA methods have been tested before in a similar experimental setup to assess their performance to produce a WGA product, adequate for CNV analysis^6^. REPLI-g single cell kit (Qiagen, Hilden, Germany) is based on this MDA mechanism and has already been shown useful for STR analysis^10^. PicoPLEX WGA Kit (Rubicon Genomics Inc., MI 48108, USA) is a hybrid MDA-PCR-based WGA method that uses self-inert degenerate primers. During the multiple displacement pre-amplification step, an *in vitro* hairpin template library is created, which is then amplified in a PCR reaction using flanking universal priming sites. PicoPLEX WGA kit has been widely used and the suitability of this kit for CNV analysis on a limited number of cells has been proven by several studies^3,11,12^. Ampli1 WGA Kit (Silicon Biosystems, Castel Maggiore, Italy) is a ligation-adapter PCR-based WGA method that utilizes a frequent cutter restriction enzyme for the initial fragmentation of the DNA template. One single highly specific primer, complementary to the adapters that are ligated on both sides of each DNA fragment, initiates the subsequent PCR amplification reaction. This ligation-based WGA method has already been successfully applied in a cbNIPT context^11^. The most recently developed DOPlify WGA (Reproductive Health Science, Thebarton, Australia) uses an advanced Degenerate Oligonucleotide Primed PCR (DOP-PCR). In the past, the classical DOP-PCR did not result in reliable CNV or STR analysis^13^. Nevertheless, DOPlify Reaction Kit is an advanced version of this classical DOP-PCR and has recently shown promising results for CNV analysis, with minimal bias introduction^6^. This kit has not yet been tested in the context of STR profiling.

Samples consisting of one or three cells, in triplicate, were collected from a B-lymphoblastoid cell line (B-LCL) using micromanipulation. As many clinical applications are based on single-cell analysis, single-cell samples are used as input in this study. Three-cell samples are included to detect possible differences in the performance of STR analysis after WGA on a few cells compared to a single cell. The lymphoblastoid cell line is representative for most human cells and is ideal as a reference for STR analysis, as it is diploid without any genomic aberrations. The samples were amplified using the four above-mentioned WGA kits and for each sample, a selection of 14 STR loci and the Amelogenin locus were amplified in a multiplex PCR reaction. A bulk DNA sample from the B-LCL was included in the STR-PCR reaction and used as a reference STR-profile against which all sample results were compared.

## Materials and Methods

### Experimental design

**Figure 1:**
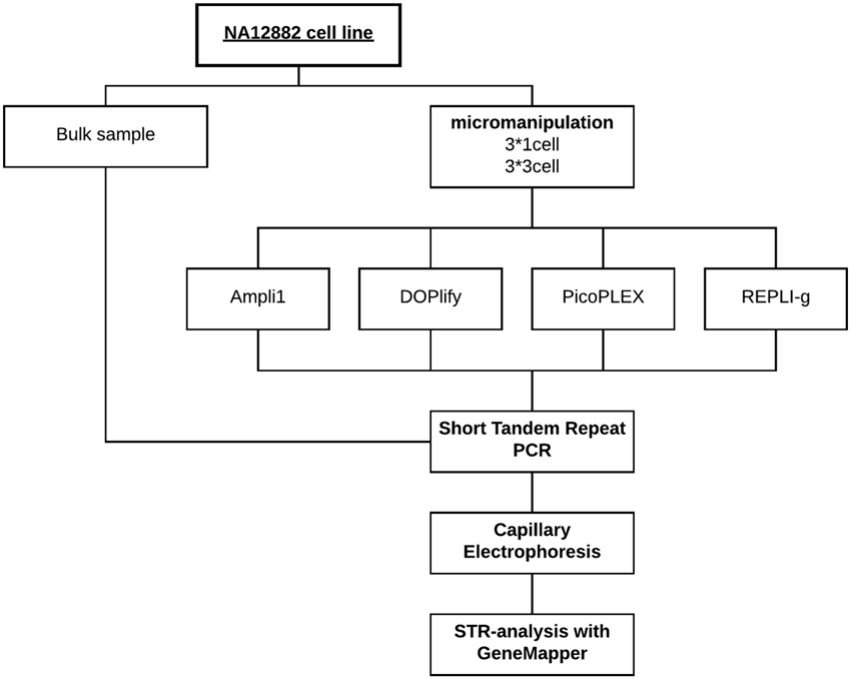
Schematic overview of the experimental design.

In this study, the performance of four WGA kits was examined for STR-genotyping on a limited number of cells from a male B-lymphoblastoid cell line (B-LCL), NA12882. This suspension cell line allowed the isolation of individual cells, using micromanipulation. Samples consisting of one or three cells, in triplicate, were collected for each WGA method. In parallel, a bulk DNA sample from the cell line was extracted. The 1- and 3-cell samples were amplified using Ampli1 WGA Kit (Silicon Biosystems, Castel Maggiore, Italy), DOPlify WGA (Reproductive Health Science, Thebarton, Australia), REPLI-g Single Cell Kit (Qiagen, Hilden, Germany) and PicoPLEX WGA Kit (Rubicon Genomics Inc., MI 48108, USA). STR-genotyping was performed on all WGA amplified DNA samples and the bulk sample. The STR-profile from the bulk DNA sample served as a reference to which all STR-profiles were compared (Fig. 1).

A positive control sample containing 1 ng male human genomic 2800 M Control DNA (Promega, Wisconsin, USA) was included during PCR to verify the success of the STR-multiplex PCR reaction. A negative control, consisting of H_2_O, was included to detect contamination introduced during the STR-PCR.

### Cell culture and isolation

The B-lymphoblastoid cell line (NA12882), acquired from Coriell Institute for Medical Research (Camden, USA), was grown in Roswell Park Memorial Insititute (RPMI-1640) medium (Life technologies, Carlsbad, USA). This cell medium was supplemented with 15% fetal bovine serum (Life Technologies, Carlsbad, USA), 2 mM L-glutamine (Life technologies, Carlsbad, USA) and a mix of penicillin at 100 units/mL and streptomycin at 100 µg/mL (Life technologies, Carlsbad, USA). Cells were cultured at a temperature of 37 °C and a 5% CO_2_ level. Cells were micromanipulated in a similar fashion as described by Deleye *et al*.^6^, except for the storing of the cells on ice after isolation. A bulk DNA sample from the NA12882 cell line was prepared using the DNeasy Blood & Tissue kit (Qiagen, Hilden, Germany) on ± 5*10^6^ cells, following manufacturer’s instructions.

### Whole genome amplification

Cell lysis and amplification using Ampli1 WGA Kit (Silicon Biosystems, Castel Maggiore, Italy), DOPlify WGA (Reproductive Health Science, Thebarton, Australia), REPLI-g Single Cell Kit (Qiagen, Hilden, Germany) and PicoPLEX (Rubicon Genomics Inc., MI 48108, USA) WGA Kit, was performed according to manufacturer’s instructions. A positive control containing ±30 pg high quality DNA (Human Genomic DNA, Roche, 100 µg (500 µl)) was included during each WGA reaction. A negative control, consisting of 1 µl of H_2_O, was added to exclude possible contamination during WGA. Purification of the amplified DNA was performed, using the Genomic DNA Clean & Concentrator kit (version 1.0.0, Zymo Research, Irvine, USA) following manufacturer’s recommendations. Qubit dsDNA High Sensitivity Assay kit (Life technologies, Carlsbad, USA) was used to determine DNA concentration.

### STR-genotyping

STR-genotyping was performed using an in-house developed multiplex STR-PCR, based on the forensic Promega Powerplex 16 kit. This multiplex-PCR was used to simultaneously amplify 14 STR loci across the human genome in each sample: D3S1358, TH01, D21S11, D18S51, vWA, D8S1179, TPOX, FGA, D5S818, D13S317, SE33, CD4, D7S820, D16S539, and the Amelogenin locus for sex determination. The PCR reaction mix contained 2.5 U Hotstar Taq polymerase (Qiagen, Hilden, Germany), 0.5 mM MgCl_2_ (Qiagen, Hilden, Germany), 0.4 µg/µL albumin (Sigma-Aldrich, Saint Louis, USA), 1× PCR buffer (Qiagen, Hilden, Germany), 0.15 µM–1 µM of each primer and 30 µL of purified DNA, in a total end volume of 50 µl. Supplementary Table S1 shows the exact concentrations used for each primer. For Ampli1, DOPlify, REPLI-g and PicoPLEX respectively 1 ng, 1 ng, 4 ng and 5 ng purified amplification product was added to the reaction mixture. The multiplex-PCR consisted of three steps: an initial denaturation step (95 °C, 15 min), 28 amplification cycli (94 °C, 1 min; 58 °C, 1 min; 72 °C, 1 min 20 s) and a final elongation step (72 °C, 10 min) and was performed in a SimpliAmp Thermal Cycler (Life Technologies, Carlsbad, USA).

### STR Genotyping analysis

STR profiles were generated with fragment size capillary electrophoresis using the ABI 3500 Genetic Analyzer equipped with GeneMapper ID-x 1.2 fragment size analysis software (Applied Biosystems, Carlsbad, USA) following manufacturer’s recommendations. Allele peaks were indicated by maintaining a detection threshold of 50 RFU. The dropout rate (DO%) was assessed for all samples and compared between the four WGA methods as well as between the 1- and 3-cell samples per WGA method. The DO% indicates how many of the expected alleles are present or missing in the sample STR profiles. The dropout rate was calculated, based on equation (1).

1 $$[1-(\frac{total\,number\,of\,observed\,alleles}{total\,number\,of\,expected\,alleles})]\times 100 \% $$

## Results and Discussion

### DNA yield

After WGA, the DNA yield was assessed and compared between the four WGA methods (Table 1). The yield was similar for Ampli1, DOPlify and PicoPlex, except for two single cell samples from Ampli1. All samples, except those two, contained ±1000 ng of DNA. The yield after REPLI-g was considerably higher than the other WGA methods. Yields after either one- or three-cell amplification do not differ significantly for all tested WGA methods.

**Table 1:** DNA yield after WGA amplification for each method.

WGA method	Sample		Concentration (ng/µL)	DNA yield (ng)
*Ampli1 WGA kit*	1-cell	1	6.14	190.34
		2	37.80	1171.80
		3	18.04	559.24
	3-cell	1	42.00	1302.00
		2	59.40	1841.40
		3	33.00	1023.00
*DOPlify reaction kit*	1-cell	1	32.60	1010.60
		2	32.60	1010.60
		3	31.60	979.60
	3-cell	1	30.60	948.60
		2	33.40	1035.40
		3	35.40	1097.40
*PicoPLEX WGA kit*	1-cell	1	60.80	1884.80
		2	52.80	1636.80
		3	54.80	1698.80
	3-cell	1	60.60	1878.60
		2	33.00	1023.00
		3	57.40	1779.40
*REPLI-g Single Cell kit*	1-cell	1	1200.00	37 200.00
		2	604.00	18 724.00
		3	690.00	21 390.00
	3-cell	1	438.00	13 578.00
		2	384.00	11 904.00
		3	516.00	15 996.00

The absence of contamination after Ampli1, DOPlify and PicoPLEX WGA was proven by the negligible DNA yield in their respective negative control. However, the negative control from REPLI-g resulted in a concentration of more than 1000 ng/µl. According to the kit’s manual, the presence of high DNA yield in negative controls is caused by the random extension of primer dimers in the absence of a template, generating high-molecular-weight product. The yield for all positive controls was similar to the yield of the cell samples of the corresponding WGA methods.

Overall, the yield after WGA did not indicate any problems during amplification and was certainly high enough to perform both STR analysis and other conceivable downstream genetic analyses.

### Dropouts in STR profiles

**Figure 2:**
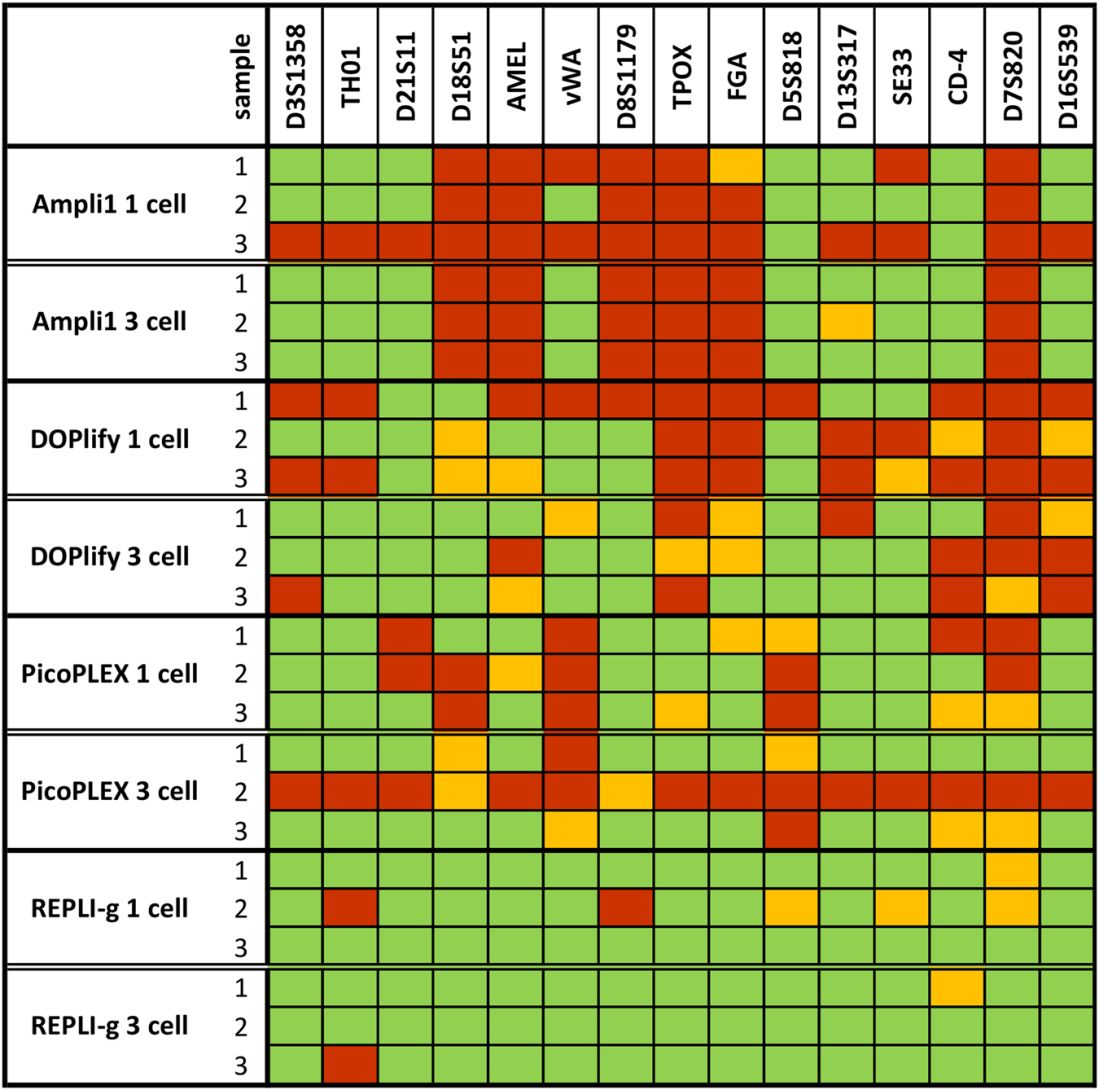
Overview of dropouts in STR profiles for each WGA method. This figure illustrates which loci were called in each sample compared to the reference profile. Green represents correctly called loci; Orange represents an allelic dropout at a heterozygous locus; Red indicates a complete locus dropout.

**Figure 3:**
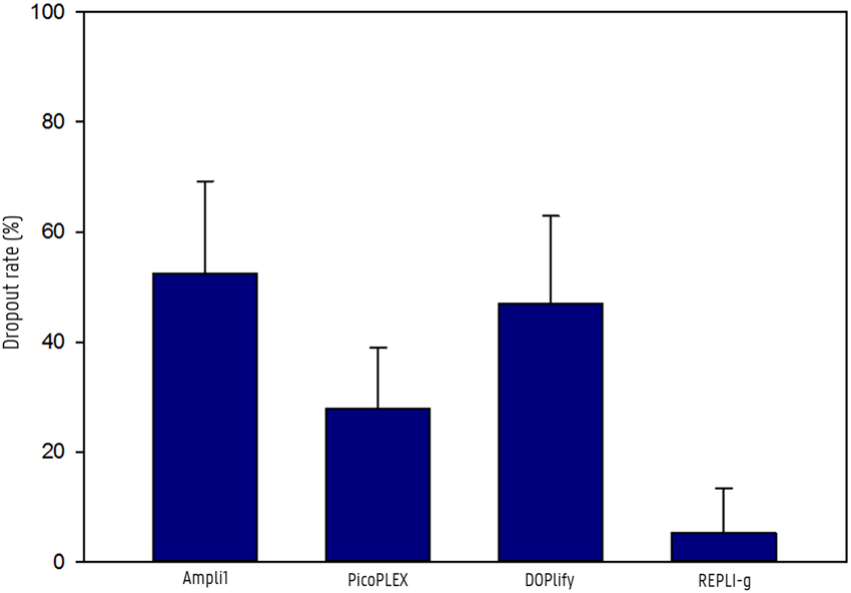
Average dropout rates per WGA method. The column graph illustrates the average dropout rates per WGA method. Error bars show the standard deviation.

The STR profiles of the samples were compared to the reference STR profile of the bulk sample from the NA12882 cell line. The reference profile and the profiles of all samples are shown in Supplementary Figure S1 and S2 respectively. Figure 2 shows for each sample which loci were called in accordance to the reference. Green represents correctly called loci, orange represents an allelic dropout at a heterozygous locus, and red indicates a complete locus dropout. The dropout rate was calculated for each sample. The average dropout rate and standard deviation for the different WGA methods is shown in Fig. 3.

For REPLI-g WGA, the generated STR profiles were almost identical to the reference profile. Consequently, the dropout rate for single- and three-cell samples amplified with REPLI-g was only 8.33% ± 11.48 and 2.38% ± 2.06, respectively. One single-cell sample showed two locus dropouts and three allelic dropouts, while the other REPLI-g samples showed no or maximum one dropout in their STR profile. The dropout rate for samples amplified with Ampli1, DOPlify and PicoPLEX was distinctly higher compared to REPLI-g. In the Ampli1 samples, the dropout seems consistent for certain loci, as a recurrent locus dropout of the same 6 loci was observed in almost all samples. No alleles were detected in all Ampli1 samples for D18S51, AMEL, D8S1179, TPOX, FGA and D7S820 with exception of one single-cell sample showing an allelic dropout for FGA instead of a locus dropout. The dropout rate for single- and three-cell Ampli1 amplification was 60.71% ± 22.30 and 44.05% ± 2.06, respectively. The dropout after PicoPLEX and DOPlify WGA seemed more random across the different loci, although some consistency among the samples was noticeable. A dropout for the vWA and D5S818 locus was observed in almost all PicoPLEX samples, whereas for DOPlify almost all samples showed a dropout for TPOX, D7S820 and D16S539. After PicoPLEX WGA, one sample failed to show an STR profile. Therefore, this sample was omitted from the dropout rate calculation for PicoPLEX. Apart from this sample, PicoPLEX samples showed less dropouts compared to both Ampli1 and DOPlify with a dropout rate of 35.71% ± 3.57 after single-cell amplification and 16.07% ± 0.00 after three-cell amplification. The dropout rate for DOPlify was 59.52% ± 12.54 for single-cell samples, whereas for three-cell samples a dropout rate of 34.52% ± 2.06 was calculated.

Previous studies already indicated that MDA is suitable for STR analysis, but no comparison has been made with other modern WGA techniques. We observed that the MDA method used in this study, REPLI-g, was the only WGA that resulted in complete STR profiles for some of its samples. Because of the branched network formation during WGA, this MDA technology amplifies more regions. Therefore, it is more likely that all STR loci are included during amplification. The recurrent locus dropout of Ampli1 may also be related to its WGA working mechanism. The restriction enzyme might cleave at the STR primer binding sites of these loci, which inhibits their amplification. Therefore, the results for Ampli1 could possibly improve if other loci were selected for STR analysis. PicoPLEX showed slightly better results than both Ampli1 and DOPlify, but the amplification protocol seems less robust, as one of its samples failed to show an STR profile. The pre-amplification during the PicoPLEX amplification protocol uses the MDA technology, which might explain why PicoPLEX is the second best WGA for STR analysis. However, PicoPLEX uses another polymerase than the one used by REPLI-g and no large, branched network is formed during pre-amplification. The four WGA methods showed a slightly better STR profile and less variability between individual samples after three-cell amplification compared to single-cell amplification.

Nevertheless, in the context of cell-based NIPT, fetal cell identification would also be possible from a partial STR profile. Therefore, Ampli1, PicoPLEX and DOPlify are also suitable for genetic identification in cell-based NIPT or other clinical genetic identification purposes, despite the fact that in most samples only half of the STR markers were detected. In cell-based NIPT or PGD, the WGA products are mainly intended for downstream genetic analysis, such as CNV detection. Therefore, beside STR analysis the WGA product must also be suitable for CNV analysis. REPLI-g showed its suitability for CNV detection on a limited number of cells at a resolution of 3 Mb earlier, but did introduce some representation bias^6^. DOPlify introduced almost no representation bias for CNV calling in that context, but its STR profiling resulted in more dropouts compared to REPLI-g. Both Ampli1 and PicoPLEX showed slightly more representation bias compared to DOPlify for CNV calling, but less compared to REPLI-g.

## Conclusion

We can conclude that DNA from a limited number of B-lymphoblastoid cells is suitable for STR profiling after WGA, but the quality of the results will depend on the selected WGA method. REPLI-g has a very low dropout rate and is the only method in this study that can provide a complete STR profile in some of its samples. Although Ampli1, DOPlify and PicoPLEX do not detect all selected STR markers, they also seem suited for genetic identification in clinical single-cell applications.

## Electronic supplementary material

Supplementary information
