# Supplementary material for: Short Tandem Repeat analysis after Whole Genome Amplification of single B-lymphoblastoid cells
Source: Sci Rep. 2018 Jan 19;8:1255. doi: 10.1038/s41598-018-19509-5 (PMC5775416; doi:10.1038/s41598-018-19509-5)

Short Tandem Repeat analysis after Whole Genome Amplification of single B-lymphoblastoid cells

Lieselot Deleye[1,#], Ann-Sophie Vander Plaetsen[1,#], Jana Weymaere[1], Dieter Deforce[1,†], Filip Van Nieuwerburgh[1,†,*]

[1]Laboratory of Pharmaceutical Biotechnology, Ghent University, Ottergemsesteenweg 460, 9000 Ghent, Belgium
[#] These authors contributed equally.
[*]Corresponding author (email: Filip.VanNieuwerburgh@UGent.be).
[†]These authors jointly supervised.

Contact information:

Prof. Filip Van Nieuwerburgh
Ghent University, Laboratory of Pharmaceutical Biotechnology
Ottergemsesteenweg 460, 9000 Ghent, Belgium
email: Filip.VanNieuwerburgh@UGent.be

Supplementary Table S1: Concentration of the STR primers in each PCR reaction mixture

Primer	Concentration	
SE33 F	0,5000	µM
SE33 B	1,0000	µM
D5S818 F	0,5000	µM
D5S818 B	0,5000	µM
FGA F	0,5000	µM
FGA B	0,5000	µM
D13S317 F	0,5000	µM
D13S317 B	0,5000	µM
vWA F	0,5000	µM
vWA B	0,5000	µM
D18S51F	0,5000	µM
D18S51 B	0,5000	µM
Amel F	0,5000	µM
Amel B	0,5000	µM
D21S11 F	0,5000	µM
D21S11 B	0,5000	µM
D3S1358 F	0,5000	µM
D3S1358 B	0,5000	µM
Tho1P16 F	0,5000	µM
Tho1P16 B	0,5000	µM
TPOX F	0,2500	µM
TPOX B	0,2500	µM
D7S820 F	0,6000	µM
D7S820 B	0,6000	µM
D16S539 F	0,8000	µM
D16S539 B	0,8000	µM
D8S1179 F	1,0000	µM
D8S1179 B	1,0000	µM
CD4 F	0,1500	µM
CD4 B	0,6000	µM

Supplementary Figure S1: STR profile from an unamplified bulk sample from the NA12882 cell line.

One peak represents a homozygous locus, whereas two peaks represent a heterozygous locus. The colored box above the peaks indicate the 14 tetrameric STR loci and the Amelogin locus.

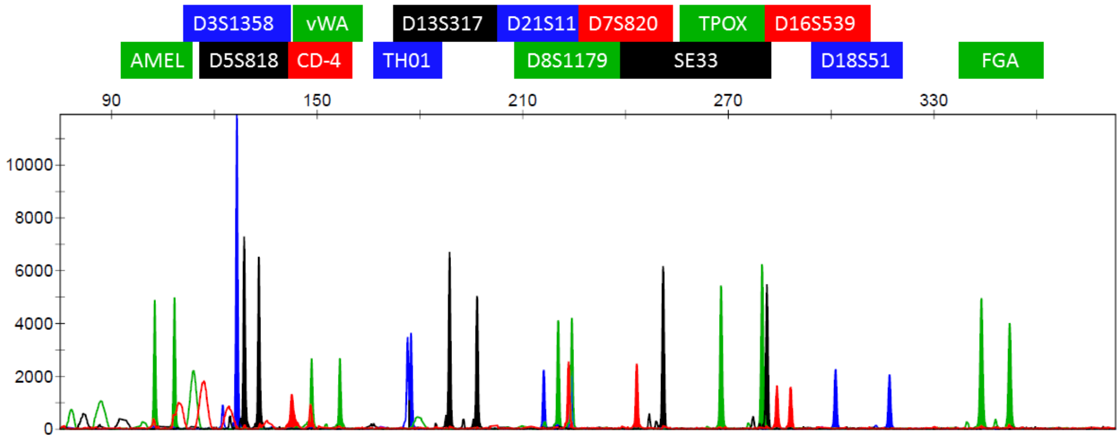

Supplementary Figure S2: STR profiles of all samples. REPLI-g STR profiles of the 1-cell samples (left) and the 3-cell samples (right).

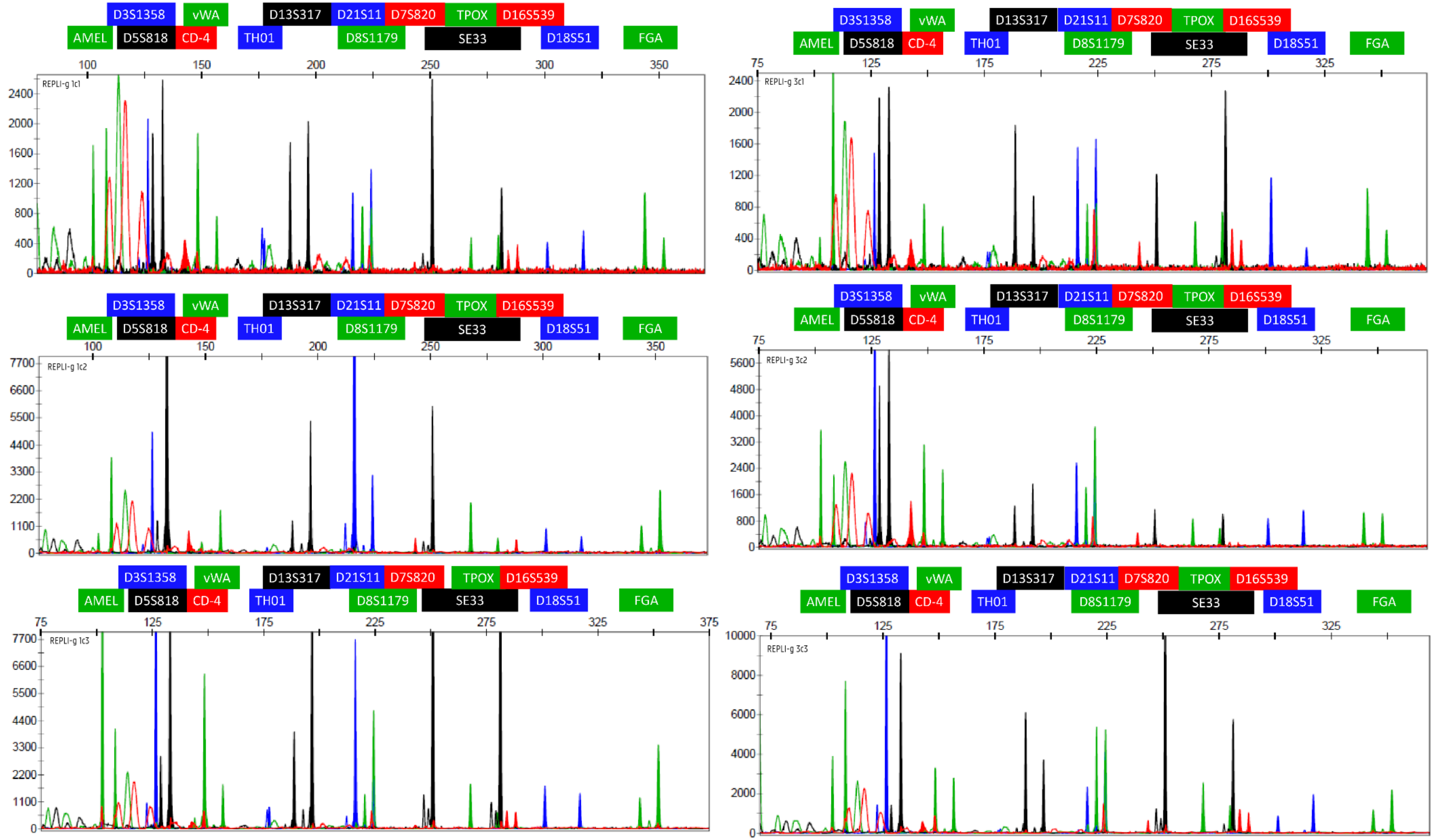

Supplementary Figure S2: STR profiles of all samples. DOPlify STR profiles of the 1-cell samples (left) and the 3-cell samples (right).

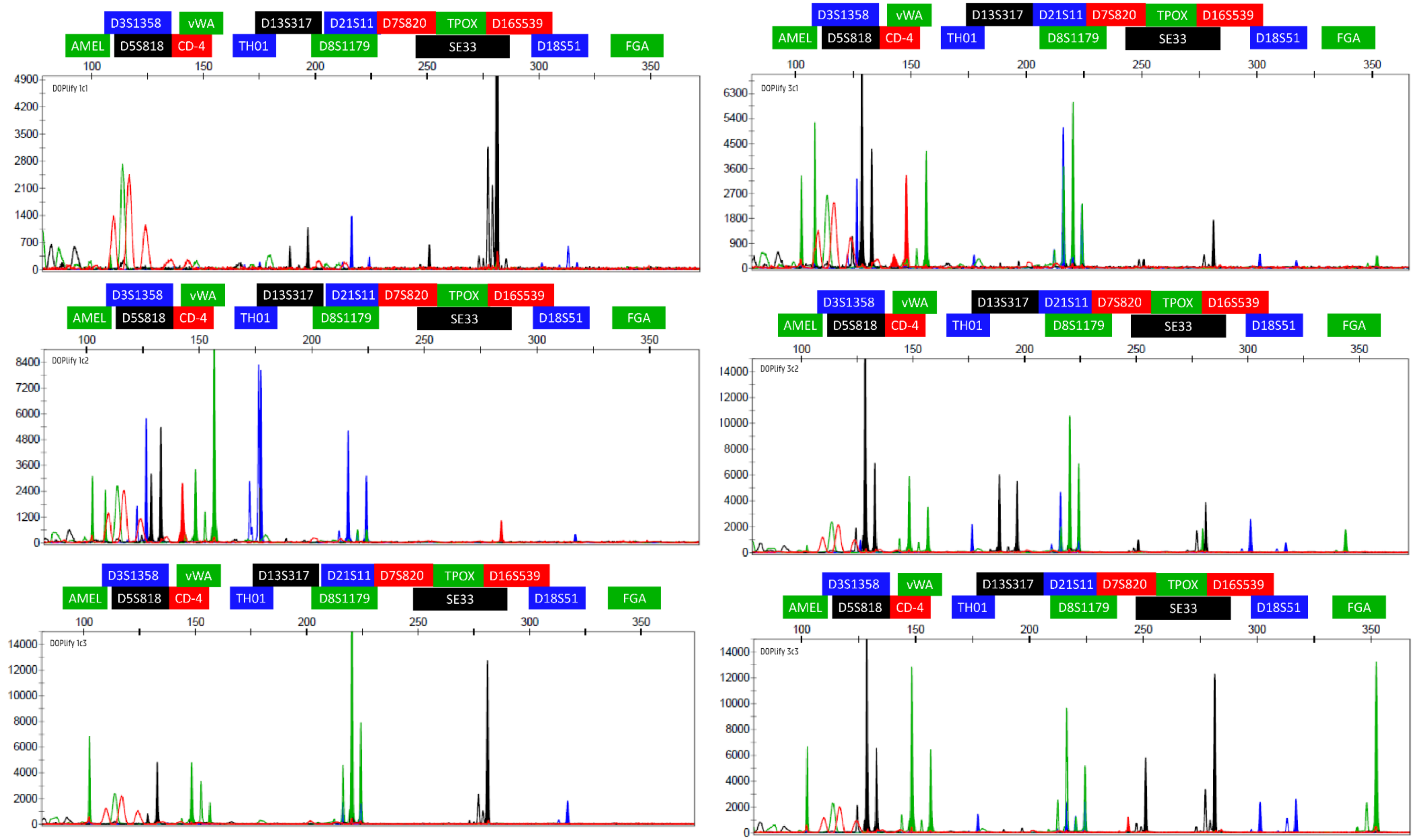

Supplementary Figure S2: STR profiles of all samples. PicoPLEX STR profiles of the 1-cell samples (left) and the 3-cell samples (right).

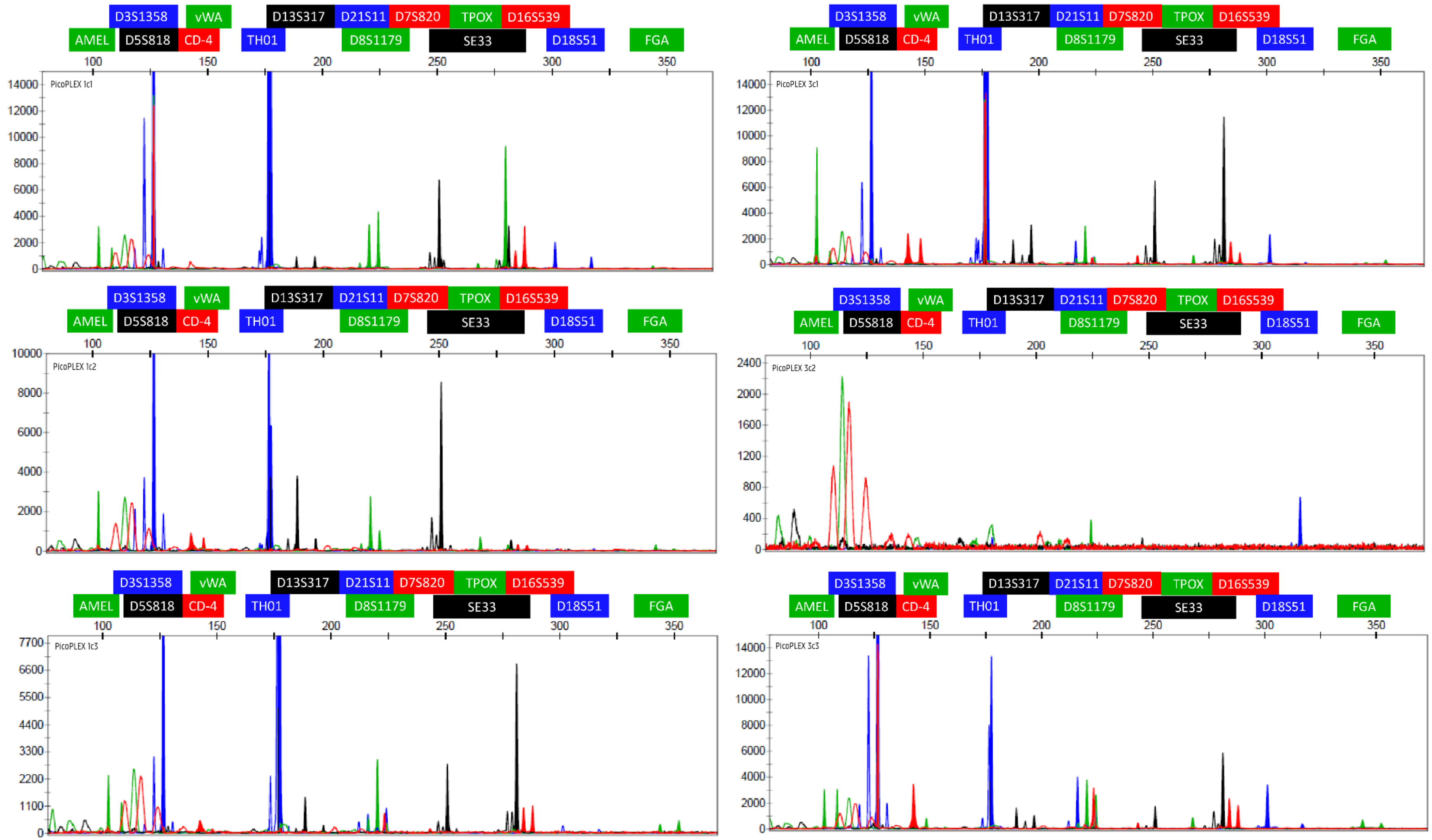

<u>Supplementary Figure S2: STR profiles of all samples.</u> Ampli1 STR profiles of the 1-cell samples (left) and the 3-cell samples (right).

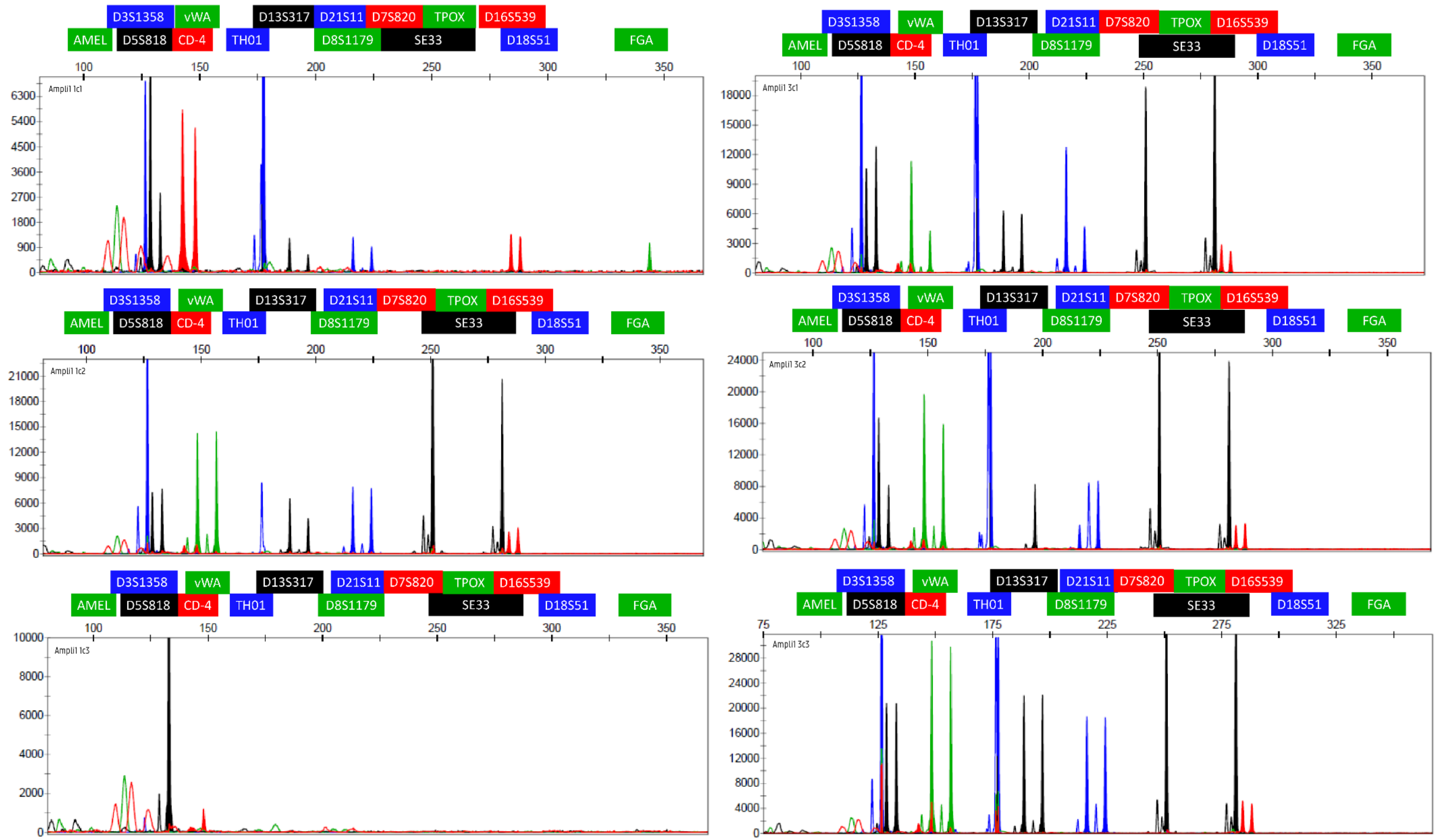

Supplement: Supplementary file 1 — Supplementary information [file 41598_2018_19509_MOESM1_ESM.pdf]
